# Icaritin Causes Sustained ERK1/2 Activation and Induces Apoptosis in Human Endometrial Cancer Cells

**DOI:** 10.1371/journal.pone.0016781

**Published:** 2011-03-08

**Authors:** Jing-Shan Tong, Qing-Hua Zhang, Xin Huang, Xue-Qi Fu, Shu-Tao Qi, Ya-Peng Wang, Yi Hou, Jun Sheng, Qing-Yuan Sun

**Affiliations:** 1 State Key Laboratory of Reproductive Biology, Institute of Zoology, Chinese Academy of Sciences, Beijing, China; 2 College of Life Sciences, Jilin University, Changchun, China; 3 Yunnan Agricultural University, Kunming, China; Health Canada, Canada

## Abstract

Icaritin, a compound from *Epimedium Genus*, has selective estrogen receptor (ER) modulating activities, and posses anti-tumor activity. Here, we examined icaritin effect on cell growth of human endometrial cancer Hec1A cells and found that icaritin potently inhibited proliferation of Hec1A cells. Icaritin-inhibited cell growth was associated with increased levels of p21 and p27 expression and reduced cyclinD1 and cdk 4 expression. Icaritin also induced cell apoptosis accompanied by activation of caspases as evidenced by the cleavage of endogenous substrate Poly (ADP-ribose) polymerase (PARP) and cytochrome c release, which was abrogated by pretreatment with the pan-caspase inhibitor z-VAD-fmk. Icaritin treatment also induced expression of pro-apoptotic protein Bax with a concomitant decrease of Bcl-2 expression. Furthermore, icaritin induced sustained phosphorylation of extracellular signal-regulated kinase1/2 (the MAPK/ ERK1/2) in Hec1A cells and U0126, a specific MAP kinase kinase (MEK1/2) inhibitor, blocked the ERK1/2 activation by icaritin and abolished the icaritin-induced growth inhibition and apoptosis. Our results demonstrated that icaritin induced sustained ERK 1/2 activation and inhibited growth of endometrial cancer Hec1A cells, and provided a rational for preclinical and clinical evaluation of icaritin for endometrial cancer therapy.

## Introduction

Endometrial cancer is one of the most common female pelvic malignancies and is the fourth most common type of cancer in North American women behind lung, breast, and colon cancers, with 42,160 new cases and 7,780 deaths estimated for 2009 [1], [2], [3]. About 81,500 women are affected every year in the European Union and the incidence is increasing. Median age of occurrence is 63 years, while >90% of women are older than 50 [4]. Although patients diagnosed with and treated for early stage-disease of the endometrioid histology enjoy relatively good survival rates, patients with advanced (stage III or IV, according to the newly revised system by the International Federation of Gynecology and Obstetrics [FIGO]) or recurrent endometrial cancer have a poor prognosis [5]. For those women with early stage disease, surgery with individualized use of volume directed radiotherapy is curative [6]. For those women with advanced stage disease, there is no real standard of care and traditionally these women are treated with surgery, chemotherapy and radiation, in one or more combinations. In the setting of advanced or recurrent disease, particularly when it is not amenable to surgical resection, the hallmark of therapy has been chemotherapy [7]. Although many patients initially respond to chemotherapy, resistance and tumor relapse eventually develop [5]. Thus, it is urgent to develop novel therapeutic agents to effectively treat this deadly disease.

**Figure 1:**
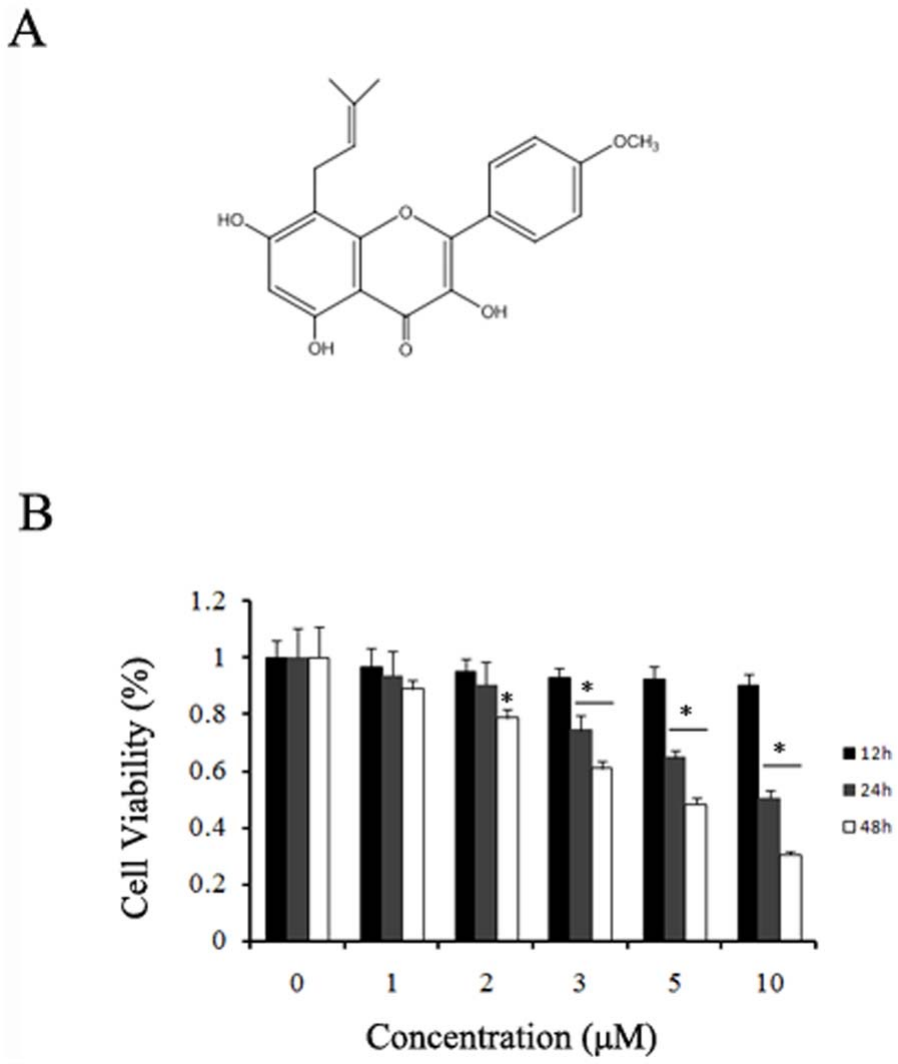
Icaritin inhibits Hec1A cells growth. (A) The chemical structure of icaritin. (B) Effects of icaritin on the growth inhibition of Hec1A cells. Cells were maintained in phenol red-free media with 2.5% charcoal-stripped fetal serum for 24 h, and then treated with the indicated concentration of icaritin. Cells were harvested at different time points as the indicated and proliferation potential was assessed by MTT assay. Results of eight independent experiments were averaged and mean ± SEM. *, *P*<0.05 compared to control cells.

Icaritin (Fig. 1A) is a hydrolytic product of icariin from *Epimedium*, a traditional Chinese herbal medicine. Icaritin exhibits many pharmacological and biological activities, such as stimulation of neuronal and cardiac differentiation [8], [9], enhancement of osteoblastic and suppressed osteoclastic differentiation and activity [10], prevention of steroid-associated osteonecrosis [11], inhibition of human prostate carcinoma PC-3 cell growth [12], induction of human prostatic smooth muscle cells apoptosis via ERK1/2 pathway [13], and neuroprotective effects [14]. Previously, it was reported that icaritin exhibits estrogen-like activity in estrogen receptor-positive breast cancer MCF-7 cells at sub-micromolar concentrations [15]. At micromolar range, however, icaritin inhibited growth of prostate cancer PC-3 cells [12]. These results indicated that icaritin has both agonist and antagonist activities depending on concentrations and may function as an estrogen receptor modulator to regulate cell growth. However, there are no reports on activity of icaritin against endometrial cancer.

Mitogen-activated protein (MAP) kinases participate in diverse cellular functions such as cell proliferation, cell differentiation, cell motility, and cell death [16]. There are three major MAPK family subgroups: extracellular signal-regulated kinase1/2 (ERK1/2), c-Jun N-terminal of stress-activated protein kinases1/2 (JNK1/2) and the p38 protein kinases. The signaling cascades involving JNK and p38, activated by extracellular stress signals, are involved in cell differentiation and apoptosis [17], [18]. Previous studies have demonstrated that transient activation of ERK1/2 plays a pivotal role in cell proliferation and that sustained ERK1/2 activation induces cell cycle arrest and differentiation [19], [20]. In the present study, we demonstrated here that activation of ERK1/2 signaling pathway mediates icaritin-induced apoptosis of Hec1A cells.

In the present study, we found that icaritin triggered a mitochondrion-mediated Hec1A cells apoptosis and sustained activation of the MAPK/ERK1/2 pathway. Our results suggested that icaritin might be useful as an anticancer agent in endometrial cancer therapy.

## Results

### Icaritin inhibits growth of endometrial cancer Hec1A cells

Previously, it was reported icaritin potently inhibited growth of prostate cancer PC-3 cells, breast cancer cells and hepatoma HepG2 cells [12], [15], [21]. We decided to examine the effect of icaritin on growth of endometrial Hec1A cells. Cells were treated with various concentrations of icaritin for 12 h, 24 h, and 48 h, and cell growth was measured with the MTT assay. We found that there was a dose-dependent and time-dependent reduction of the growth of Hec1A cells treated with icaritin (Fig. 1B), indicating that icaritin inhibits proliferation of Hec1A cells.

**Figure 2:**
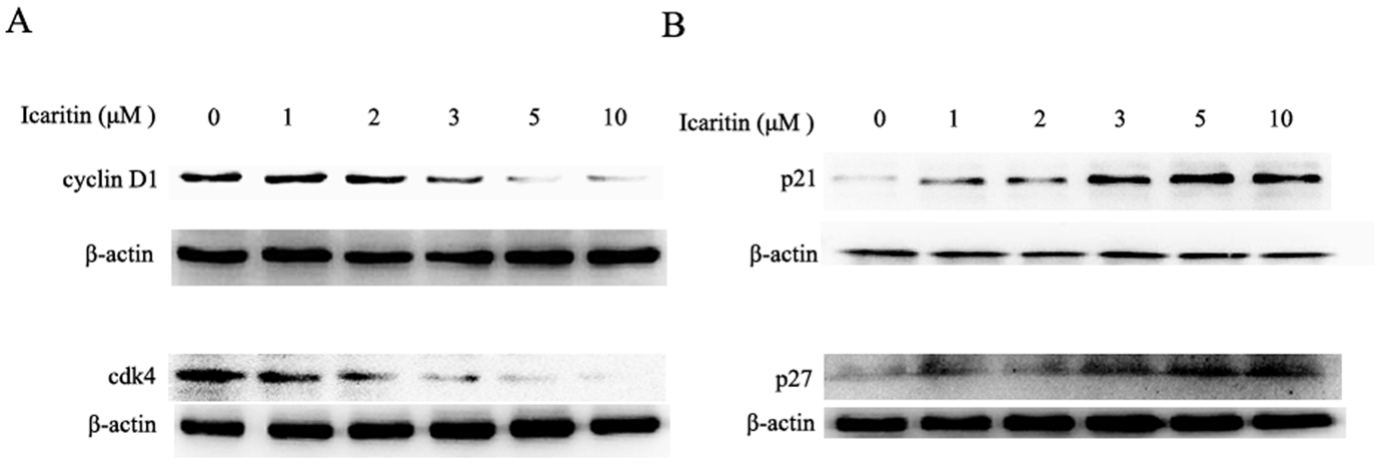
Effects of icaritin on cell cycle regulators in Hec1A cells. (A) Hec1A cells were treated with the indicated concentration of icaritin for 24 h. The levels of cyclin D1 and cdk4 were determined by western blot. Protein levels of β-actin were also measured as controls. (B) Hec1A cells were treated with the indicated concentration of icaritin for 24 h. The levels of p21 and p27 were determined by western blot. Protein levels of β-actin were also measured as controls.

To probe the mechanism underlying icaritin-induced cell cycle arrest, major G1 phase regulators, such as cyclin D1/cdk4 and cyclin-dependent kinase inhibitors WAF1/p21 and KIP1/p27 were examined. As shown in Fig. 2A, icaritin treatment resulted in a marked decrease of the expression levels of cyclin D1 and cdk4 and increase of WAF1/p21 and KIP1/p27 expression compared to cells treated with vehicle (Fig. 2B). These data indicated that icaritin was able to induce cell cycle arrest and to regulate cycle-related effectors.

### Icaritin induces Hec1A cells apoptosis

**Figure 3:**
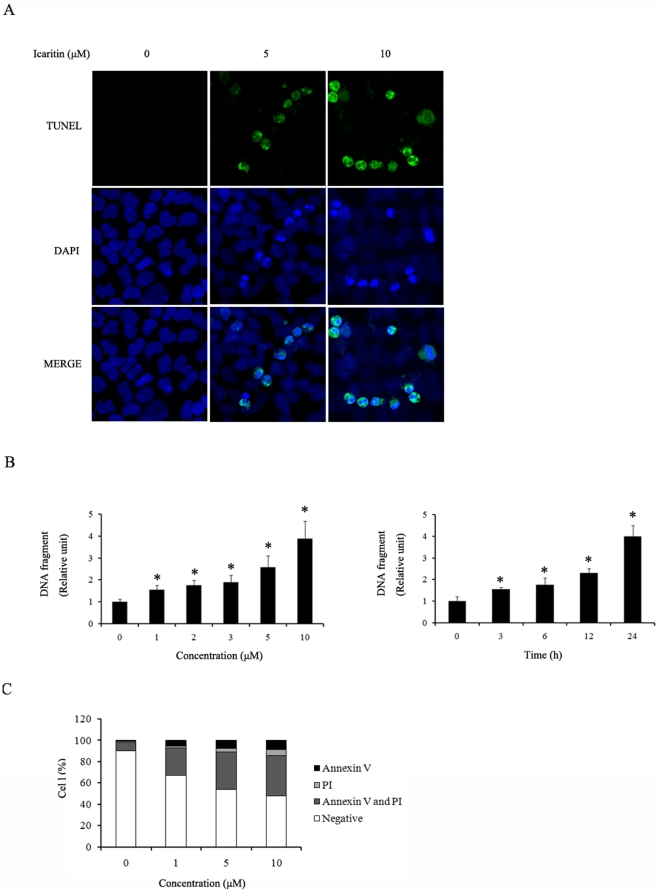
Icaritin induced Hec1A cells apoptosis. (A) Visualization of apoptotic cells by the TUNEL assay on Hec1A cells. (B) DNA fragmentation was evaluated using a Cell Death Detection ELISA kit. The data are expressed as mean ± SEM of three separate experiments. *, *P*<0.05 compared to control cells. (C) The apoptotic status was determined by Annexin V/PI staining method. Percentages of negative (viable) cells, annexin V-positive (early apoptotic) cells, PI-positive (necrotic) cells, or annexin V and PI double-positive (late apoptotic) cells were shown (mean of three independent experiments) by a flow cytometry analysis.

We next test whether icaritin also induces apoptotic cell death in human endometrial cancer Hec1A cells. Hec1A cells were treated with icaritin and apoptosis was assayed by two different methods. As shown in Fig. 3A, the number of TUNEL-positive cells increased with increased concentrations of icaritin. Nucleosome fragmentation, an indicator of apoptosis, determined with the Cell Death Detection ELISA further confirmed that icaritin induced cell apoptosis at a dose- and time-dependent manner (Fig. 3B). In addition, an annexin V-staining also demonstrated that icaritin induced apoptotic cell death but not necrosis in Hec1A cells (Fig. 3C).

**Figure 4:**
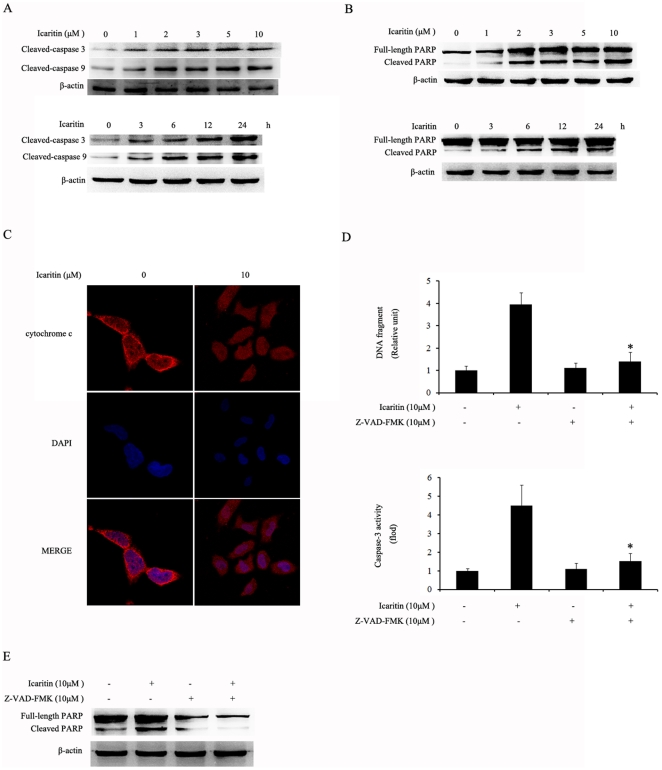
Induction of caspases activities by icaritin. (A) Hec1A cells were treated with the indicated icaritin concentration or the indicated time points, total cellular extracts were prepared and subjected to Western blot assay to measure levels of cleaved-caspase-3 and cleaved-caspase-9. Protein levels of β-actin were also measured as controls. (B) Hec1A cells were treated with the indicated icaritin concentration or the indicated time points, total cellular extracts were prepared and subjected to Western blot assay using antibodies against PARP. Protein levels of β-actin were also measured as controls. (C) Hec1A cells were fixed and labeled for cytochrome c (red) and DNA (blue). (D) Hec1A cells were pretreated with 10 µM of z-VAD-fmk for 1 h, followed with 10 µM icaritin for 24 h, and DNA fragmentation and caspase-3 activity were determined. The data are expressed as mean ± SEM of three separate experiments. *, *P*<0.05 compared to control cells. (E) Hec1A cells were pretreated with 10 µM of z-VAD-fmk for 1 h, followed with 10 µM icaritin for 24 h. Protein extracts were prepared and subjected to Western blot assay using antibody against PARP. Protein levels of β-actin were also measured as controls.

Dose- and time-dependent effects of icaritin on the proteolytic activation of caspase-3 and caspase-9 were also examined. As shown in Fig. 4A, icaritin treatment resulted in a significant increase in the active form of caspase-3 and caspase-9 in Hec1A cells. The activation of caspases in icaritin treated Hec1A cells was further confirmed by detecting the cleavage of PARP, an endogenous substrate of activated caspase-3 and a hallmark of apoptosis. As shown in Fig. 4B, treatment of Hec1A cells with icaritin resulted in cleavage of PARP to an 85 kDa fragment. In addition, we examined the subcellular localization of cytochrome c to determine whether the mitochondria pathway is involved in icaritin-induced apoptosis. Immunofluorescence of cytochrome c was visualized with a confocal laser microscope. After cells were treated with 10 µM icaritin for 24 h, the staining pattern of cytochrome c became diffuse and blurred (Fig. 4C) in contrast to the compact, plaque-like appearance of cytochrome c in the control cells treated with vehicle, indicating the release of cytochrome c from the mitochondria into the cytosol in icaritin-treated cells.

To further determine the role of caspase activation in icaritin-induced apoptosis, we treated Hec1A cells with the pan-caspase inhibitor z-VAD-fmk (10 µM) before icaritin treatment. The pan-caspase inhibitor z-VAD-fmk pretreatment abolished caspase-3 activity and reduced icaritin-induced apoptosis as measured by the nucleosome fragmentation in Hec1A cells (Fig. 4D & E). These results strongly suggest that activation of the caspase cascade was essential for icaritin-induced apoptosis in Hec1A cells.

### Icaritin regulates the expression of Bcl-2 family proteins in Hec1A cells

**Figure 5:**
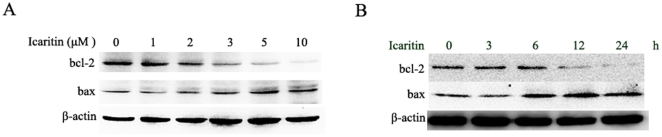
Effects of icaritin on the expression of Bcl-2 family. (A) Hec1A cells were treated with the indicated concentration of icaritin, total cellular extracts were prepared and subjected to Western blot assay to measure levels of Bcl-2 and Bax. Protein levels of β-actin were also measured as controls. (B) Hec1A cells were treated with the indicated icaritin time points of icaritin, total cellular extracts were prepared and subjected to Western blot assay to measure levels of Bcl-2 and Bax. Protein levels of β-actin were also measured as controls.

The anti-apoptotic Bcl-2 is a potent antagonist of the mitochondrial pathway of apoptosis initiated by a variety of extra- and intracellular stresses. We decided to test the effects of icaritin on the expression of anti-apoptotic protein Bcl-2 and the pro-apoptotic proteins Bax in the Hec1A cells with the Western blot analysis. As shown in Fig. 5, icaritin increased in the levels of Bax expression while reduced Bcl-2 expression at a dose- and time- dependent manner, indicating that icaritin also regulates expression levels of the Bcl-2 family proteins to facilitate apoptotic cell death induced by icaritin.

### Icaritin induces sustained ERK1/2 activation in Hec1A cells

**Figure 6:**
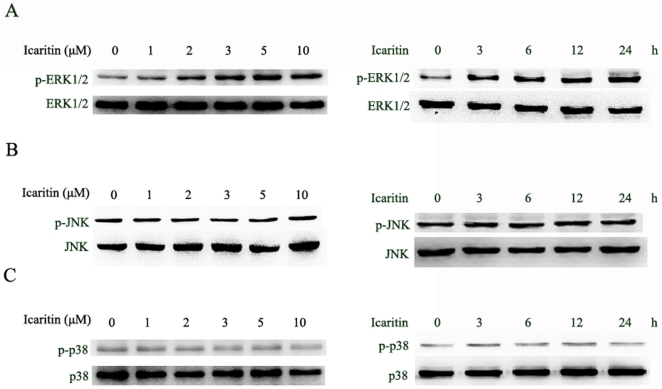
Effects of icaritin on MAPK pathways. Hec1A cells were treated with the indicated icaritin concentration or the indicated interval, total cellular extracts were prepared and subjected to Western blot assay to measure levels of phosphorylated forms of ERK1/2 (A), JNK (B) and p38 (C). Membranes were reprobed with antibodies against total ERK1/2, JNK and p38 for normalization.

Cellular stresses and stimuli induce cell apoptosis via sustained activation of the MAPK signaling pathways [22], [23]. We thus examined the activation of MAPKs ERK1/2, JNK and p38 after icaritin treatment. As shown in Fig. 6A, the phosphorylation levels of ERK1/2 were increased after the icaritin treatment, which last twenty-four hours. However, no significant changes of expression and phosphorylation levels of p38 and JNK were observed after icaritin treatment (Fig. 6B & C). These results suggested that sustained activation of the MAPK/ERK is involved in icaritin-induced growth inhibition and apoptosis in HecA1 cells.

### The ERK1/2 signaling pathway is involved in icaritin-induced apoptosis in Hec1A

**Figure 7:**
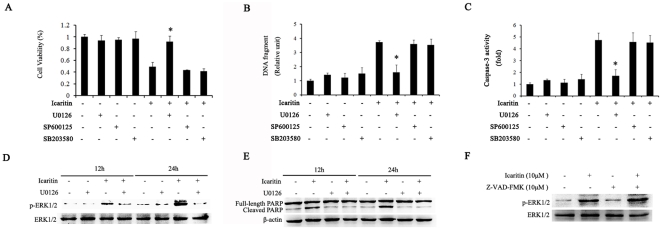
Icaritin induced Hec1A cells apoptosis was mediated through ERK1/2 activation. (A) Hec1A cells were treated with icaritin in the presence or absence of 10 µM U0126, 10 µM SP600125, or 40 µM SB203580 for 24 h, Cell growth was determined by MTT. The bars represent the mean ± SEM of three separate experiments. *, *P*<0.05 compared for the icaritin-treated group. (B) Hec1A cells were treated with icaritin in the presence or absence of 10 µM U0126, 10 µM SP600125, or 40 µM SB203580 for 24 h, and DNA fragmentation was determined. The data are expressed as mean ± SEM of three separate experiments. *, *P*<0.05 compared to icaritin-treated cells. (C) Hec1A cells were treated with icaritin in the presence or absence of 10 µM U0126, 10 µM SP600125, or 40 µM SB203580 for 24 h, and caspase-3 activity was determined by caspase-Glo assay. The data are expressed as mean ± SEM of three separate experiments. *, *P*<0.05 compared to icaritin-treated cells. (D) Hec1A cells were pretreated or not pretreated with 10 µM U0126 then added with DMSO (vehicle) or 10 µM icaritin for 12 h, 24 h respectively. Protein extracts were prepared and subjected to Western blot assay to measure levels of phosphorylated ERK1/2. Protein levels of ERK1/2 were also measured as controls. (E) Hec1A cells were pretreated or not pretreated with 10 µM U0126 then added with DMSO (vehicle) or 10 µM icaritin for 12 h, 24 h respectively. Protein extracts were prepared and the cleavage of PARP was analyzed with Western bolt. Protein levels of β-actin were also measured as controls. (F) Hec1A Cells were pretreated with or without z-VAD-fmk (10 µM) were further incubated with 10 µM icaritin for 24 h. Protein extracts were prepared and subjected to Western blot assay to measure levels of phosphorylated ERK1/2. Protein levels of ERK1/2 were also measured as controls.

We than examined whether the icaritin-induced sustained activation of the ERK1/2 signaling pathway plays a role in growth inhibition and apoptosis. As shown in Fig. 7A & B, icaritin induced cell apoptosis was effectively abrogated by U0126, a potent inhibitor of MEK1/2, but the p38 inhibitor SB203580 and JNK inhibitor SP600125 had no effect, suggesting that the activation of ERK1/2 signaling is involved the icaritin-induced apoptosis. Similarly, ERK1/2 inhibition by its specific inhibitor could effectively antagonize icaritin-induced caspase-3 activity (Fig. 7C). In addition, Western blot assay revealed that U0126 inhibited sustained ERK1/2 activation and cleavage of PARP in icaritin treated (Fig. 7D) while the caspase inhibitor z-VAD-fmk had no effect on icaritin-induced activation of ERK1/2 (Fig. 7E). These results demonstrated that the pro-apoptotic effects of icaritin in Hec1A cells are mediated by the sustained activation of the ERK1/2 signaling pathway.

## Discussion

Here, we demonstrated that icaritin, a compound purified from medicinal herb Epimedium, induces growth inhibition and apoptotic cell death in human endometrial cancer Hec1A cells at a dose and time dependent manner.

It is well established that cell cycle progression is dynamically and strictly regulated by complexes containing cdks and cyclins all of which are critical for the normal progression of cell cycle and inactivation of these proteins leads to cell cycle arrest [24], [25], [26]. The observed inhibitory effects of icaritin on the expression of cyclin D1 and cdk4 in Hec1A cells suggested that icaritin may arrest the cell cycle at a specific phase. Cdk activity is also regulated by the cdk inhibitors such as WAF1/p21 and KIP1/p27 families of proteins. Here, we also showed that icaritin induced the expression levels of the WAF1/p21 and KIP1/p27.

In many cell types, apoptosis is characterized by the generation of DNA fragments through the action of endogenous endonucleases [27], [28]. The DNA of apoptotic cells is cleaved into multimers of 180–200 bp fragments, corresponding to the oligonucleosomal size. Therefore, the DNA of apoptotic cells typically migrates as a ladder of 180–200 bp multimers on an agarose gel. The Terminal deoxynucleotidy Transferase Biotin-dUTP Nick End Labeling (TUNEL) method identifies apoptotic cells in situ by using terminal deoxynucleotidyl transferase (TdT) to transfer biotin-dUTP to these strand breaks of cleaved DNA [29]. The ELISA assay determination of the cytoplasmic histone-associated DNA fragments (mono- and oligonucleosomes) after induced cell death [30]. Annexin V is used to quantitatively determine the percentage of cells within a population that are actively undergoing apoptosis. It relies on the property of cells to lose membrane asymmetry in the early phases of apoptosis. In apoptotic cells, the membrane phospholipid phosphatidylserine (PS) is translocated from the inner leaflet of the plasma membrane to the outer leaflet, thereby exposing PS to the external environment. Annexin V is a calcium-dependent phospholipid-binding protein that has a high affinity for PS, and is useful for identifying apoptotic cells with exposed PS. Propidium Iodide (PI) is a standard flow cytometric viability probe and is used to distinguish viable from nonviable cells. Viable cells with intact membranes exclude PI, whereas the membranes of dead and damaged cells are permeable to PI. Cells that are considered viable are annexin V and PI negative; cells that are in early apoptosis are annexin V positive and PI negative; cells that are in late apoptosis are both annexin V and PI positive; and cells that are in necrotic are annexin V negative and PI negative [31]. The annexin V/PI staining method distinguishes apoptosis cells and necrosis cells. In the present study, Hec1A cells were treated with icaritin and apoptosis was assayed by using TUNEL and ELISA assay. Furthermore, an annexin V-binding assay showed that icaritin treatment induced apoptosis but not necrosis in Hec1A cells.

Mitochondria play a pivotal role in the signal transduction of apoptosis [32]. The observation of icaritin-mediated activation of caspase-9, caspase-3, subsequent cleavage of PARP and release of cytochrome c, as well as the result that the pan-caspase inhibitor z-VED-FMK almost completely blocked icaritin-induced apoptosis in Hec1A cells, suggesting that mitochondrial-mediated caspase cascade pathway plays a very important role in icaritin-induced apoptosis [33].

Bcl-2 family of proteins, including Bcl-2 and Bcl-2-related family members such as Bcl-xL, Bad and Bax, plays an important role in the regulation of apoptosis. They can activate or inhibit the release of downstream factors such as cytochrome c which leads to the activation of caspase-3 and PARP in the execution of apoptosis [34]. Bax exerts pro-apoptotic activity by translocation from the cytosol to the mitochondria, where it induces cytochrome c release, while Bcl-2 exerts its anti-apoptotic activity, at least in part, by inhibiting the translocation of Bax to the mitochondria [35]. Obviously, the ratio of pro- and anti-apoptotic protein expression, such as Bax/Bcl-2, is critical for the induction of apoptosis, and it decides the susceptibility of cells to undergo apoptosis [36]. In the present study, we showed that treatment of the Hec1A cells with icaritin resulted in significant decrease in the Bcl-2 protein and increase in the Bax protein, thus shifting the Bax/Bcl-2 ratio in favor of apoptosis. Taken together, our results indicated that icaritin regulates expression levels of the Bcl-2 family, induces cytochrome c release and trigs caspase-dependent cell apoptotic death.

The family of MAPKs, ERK, SAPK/JNK1/2 and p38, plays central roles in cell proliferation, differentiation, survival, and apoptosis, including extracellular-regulated kinase1/2 [37], [38]. In general, transient ERK1/2 activation occurs rapidly and decline with 30–45 min, which is considered to lead to cell proliferation and survival [39], but persistent or sustained ERK1/2 activation that last more than 12 h is involved in cell differentiation and death [40], [41], [42], [43]. The dual activities of ERK1/2 in both cell proliferation and cell death may be explained by several recent findings that demonstrate that phosphorylated ERKs may produce different outcomes, depending on the duration of ERK accumulation in the nucleus [44], [45]. Recently, it has been reported that sustained ERK activation was involved in calcium-induced apoptosis of lens epithelial cells [46]. Several studies have shown that icaritin induces activation of ERK and p38 kinase in embryonic stem cells and neuronal cells [8], [14]. Chen et al. reported that icaritin induces growth inhibition and apoptosis of human prostate cancer PC-3 cells through the ERK1/2 signaling pathway [13]. Here we also found that icaritin induced sustained activation of the ERK1/2, but not JNK and p38 in Hec1A cells. U0126, a specific inhibitor of MEK (the MAPK/ERK kinase), effectively blocked icaritin-induced ERK1/2 activation and attenuated icaritin-induced apoptosis, suggesting that the sustained activation of the ERK1/2 signaling pathway is one of the mechanisms.

In conclusion, we found that icaritin inhibited cell growth and induced cell apoptosis in Hec1A cells. We also studied the underlying mechanisms involved in icaritin-induced apoptosis. Our results indicated that icaritin-induced cell growth inhibition involves the reductions of cyclin D1 and cdk4 protein expression and inductions of p21 and p27 protein expression. Our results also demonstrated that icaritin-induced apoptosis involves the activation of caspase-3 and caspase-9 and release of cytochrome c. We found that sustained ERK1/2 activation was required for icaritin-induced apoptosis. Our results provided a rational that icaritin could be developed as a potential anticancer agent against human endometrial cancer.

## Materials and Methods

### Materials and Reagents

Icaritin with a purity of up to 99.5% was from Dr. Kun Meng (shenogen Pharma Co Beijing, China). A stock solution (10 µM) was prepared by dissolving icaritin in DMSO (sigma, St Louis, MO, USA) and stored at −20°C. MTT, DAPI, U0126, SP600125, SB203580, and pan-caspase inhibitor (z-VAD-fmk) were purchased from Calbiochem (San Diego, CA). Antibody for cleaved caspase-3 was purchased from Cell Signaling (Beverly, MA). Antibodies against ERK1/2, phospho-ERK1/2, p38, phospho-p38, JNK, phospho-JNK, p21, p27, cytochrome c, Bcl-2, Bax, cleaved caspase-9, cyclin D1, cdk4, PARP and β-actin were purchased from Santa Cruz Biotechnology (Santa Cruz, CA). FITC Annexin V Apoptosis Detection Kit I was purchased from Becton Dickinson (PharMingen).

### Cells culture and cell proliferation essay

The human endometrial cancer cell line Hec1A was obtained from Dr. Li-Hui Wei (Peking University People's Hospital, Beijing) and cultured in Dulbecco's modified Eagle's medium (Gibco-BRL, USA) medium with 10% fetal calf serum (Hyclone, UT), 5 ug/ml insulin, and maintained at 37°C in a humidified atmosphere of 5% CO_2_. Media will be changed in phenol red-free media with 2.5% charcoal-stripped fetal calf serum for 24 h before experiments needed.

Cells were seeded in a 96-well dish to a final concentration of 1×10^4^ cells/well and incubated in DMEM medium containing 10% FCS for 24 h. Then Cells were cultured in phenol-red-free DMEM (Gibcol-BRL, USA) with 2.5% charcoal-stripped fetal calf serum (Biochrom AG, Germany) followed by treatment with varying concentrations of icaritin for 12 h, 24 h, and 48 h. Medium was removed and fresh medium was added to each well along with 20 µl of MTT solution (5 mg/ml). After 4 h incubation, 150 µl of DMSO were added to each well. The plates were read at wavelength of 490 nm using a microplate reader (Bioteck Powerwave^TM^, USA). Eight reduplicate wells were used for each treatment, and experiments were repeated three times.

### Western Blot Analysis

Western blot was performed as described previously [47], [48]. Cells were treated with the indicated concentration of icaritin for the indicated time in phenol-red-free DMEM (Gibcol-BRL, USA) with 2.5% charcoal-stripped fetal calf serum (Biochrom AG, Germany). The cells were collected in ice-cold PBS, and the cell extracts were prepared in RIPA buffer with proteinase inhibitor cocktail from Sigma (St.Louis, MO). The protein concentrations of the cell lysates were determined and boiled with gel-loading buffer for 10 min at 100°C. Samples containing 30 µg of total protein were electrophoresed on 10% SDS-polyacrylamide gels and transferred to PVDF membrane (Millipore, Temecula, CA). Following transfer, the membrane were blocked in TBST (TBS containing 0.1% Tween 20) containing 5% skimmed milk for 2 h, followed by incubation overnight at 4°C with appropriate primary antibodies. After washing three times in TBST, the membranes were incubated for 1 h at 37°C with 1∶2000 horseradish peroxidase-conjugated appropriate secondary antibodies. Finally, the membranes were visualized using the enhanced chemiluminescence detection system (Amersham, Piscataway, NJ).

### TUNEL Assay

Potential DNA fragmentation was examined with fluorescence staining by the TUNEL (Terminal Transferase dUTP Nick End Labeling) apoptosis detection kit (Beyotime Biotech, China) following the manufacturer's instruction. Briefly, cells cultured on sterile glass coverlips were fixed with 4% paraformaldehyde in PBS for 10 min, and then permeabilized with 0.4% Triton X-100 for 10 min at room temperature. Cells were incubated with a reaction mix containing biotin-dUTP and the terminal deoxynucleotidyl transferase (TdT) for 60 min and then with the avidin-FITC solution for 30 min in the dark. DAPI was subsequently added for nuclear staining. Microscopic analysis was performed using a confocal laser-scanning microscope (Zeiss LSM 710 META, Germany).

### Apoptosis Detection by Cell Death Enzyme-Linked Immunoabsorbent Assay Method

For ELISA, the cells seeded in 96-well plates (1×10^4^ cells/well) were treated with icaritin, then apoptotic cells were evaluated by using Cell Death Detection ELISA kit (Roche Diagnostics, Mannhein, Germany) according to the manufacturer's instruction. Photometric enzyme immunoassay was used to quantitatively determine the formation of cytoplasmic histone-associated DNA fragments in the form of mononucleosomes after apoptosis of the cells. Absorbance at 405 nm was measured as the indicator of apoptotic cells. The reference wavelength was 490 nm. The enrichment factor (total amount of apoptosis) was calculated by dividing the absorbance of the sample (A405 nm) by the absorbance of the controls without treatment (A490 nm).

### Annexin V/PI assays for apoptosis

For Annexin V/PI assays, cells were stained with Annexin V-FITC and PI, and evaluated for apoptosis by flow cytometry according to the manufacturer's protocol (BD PharMingen, San Diego, CA, USA). Briefly, 1×10^6^ cells were washed twice with PBS, and stained with 5 µl of Annexin V-FITC and 10 µl of PI (5 µg/ml) in 1 ml binding buffer for 15 min at room temperature in the dark. The apoptotic cells were determined using a Becton-Dickinson FACScan cytoflurometer (Mansfield, MA, USA).

### Immunofluorescence staining

Immunofluorescence staining was used to analyze the Subcellular distribution of cytochrome c in Hec1A cells induced by Icaritin. Cells cultured on sterile glass coverlips were fixed with 4% paraformaldehyde in PBS for 10 min. After permeabilized with 0.4% Triton X-100 for 10 min at room temperature, cells were blocked in 4% BSA-supplemented PBS for 1 h and incubated overnight at 4°C with anti-cytochrome c antibody. After washing three times in PBS, cells were labeled with TRITC-conjugated secondary antibody. DAPI was subsequently added for nuclear staining. Microscopic analysis was performed using a confocal laser-scanning microscope (Zeiss LSM 710 META, Germany).

### Measurement of caspase-3 activity

Cells were treated with icaritin in the absence and presence of different kinase inhibitors, and the caspase-3 activity in the cleared lysates were measured by using Caspase-3 Activity Assay Kit (Beyotime Biotech, China) according to the manufacturer's instruction. Luminescence was quantified using an ELISA reader. Blank values were subtracted, and increases in caspase-3 activities were expressed as fold increase and calculated based on activities measured from untreated cells. Each sample was measured in triplicates.

### Statistical analysis

Statistical analysis was performed with the paired-samples *t*-test, or ANOVA followed by the Student-Newman-Keuls testing to determine differences in means. A level of *P*<0.05 was considered statistically significant. All statistical tests were three-sided.
